# Theta/beta ratio in EEG correlated with attentional capacity assessed by Conners Continuous Performance Test in children with ADHD

**DOI:** 10.3389/fpsyt.2023.1305397

**Published:** 2024-01-19

**Authors:** Tzong-Shi Wang, Syu-Siang Wang, Chang-Li Wang, Shi-Bing Wong

**Affiliations:** ^1^Department of Psychiatry, Taipei Tzu Chi Hospital, Buddhist Tzu Chi Medical Foundation, New Taipei City, Taiwan; ^2^Department of Electrical Engineering, Yuan Ze University, Taoyuan, Taiwan; ^3^Taipei Municipal Jianguo High School, Taipei, Taiwan; ^4^Department of Pediatrics, Taipei Tzu Chi Hospital, Buddhist Tzu Chi Medical Foundation, New Taipei City, Taiwan; ^5^School of Medicine, Tzu Chi University, Hualien, Taiwan

**Keywords:** quantitative electroencephalography, theta/beta ratio, Connor's Continuous Performance Test, attention deficit/hyperactivity disorder, attention capacity

## Abstract

**Introduction:**

Attention-deficit/hyperactivity disorder (ADHD) is a prevalent neurodevelopmental disorder affecting children worldwide; however, diagnosing ADHD remains a complex task. Theta/beta ratio (TBR) derived from electroencephalography (EEG) recordings has been proposed as a potential biomarker for ADHD, but its effectiveness in children with ADHD remains controversial. Behavioral assessments, such as the Conners Continuous Performance Test–3^rd^ edition (CPT-3), have been utilized to assess attentional capacity in individuals with ADHD. This study aims to investigate the correlation between TBR and CPT-3 scores in children and adolescents with ADHD.

**Methods:**

In a retrospective analysis, we examined patients regularly monitored for ADHD at Taipei Tzu Chi Hospital, who underwent both EEG and CPT-3 assessments. Severity of ADHD was evaluated using parent- and teacher-completed Swanson, Nolan, and Pelham (SNAP)-IV rating scales.

**Results:**

The study encompassed 55 ADHD patients (41 with abnormal CPT-3 scores, 14 with normal CPT-3 scores) and 45 control subjects. TBR demonstrated elevation in ADHD patients with abnormal CPT-3 scores, indicating its potential to represent attentional capacity akin to behavioral assessments like CPT-3. However, significant correlations between TBR values and CPT-3 variables or SNAP-IV rating scales were not observed. Moreover, TBR values exhibited considerable overlap across the groups, leading to diminished sensitivity and negative predictive value as a potential neurophysiological ADHD biomarker.

**Discussion:**

While our study underscores the utility of both TBR and CPT-3 in assessing attentional capacity, their sensitivity in diagnosing ADHD is limited. A comprehensive evaluation, integrating clinical expertise, parental input, and detailed neuropsychometric tests, remains pivotal for a thorough and precise diagnosis of ADHD.

## 1 Introduction

Attention-deficit/hyperactivity disorder (ADHD) is a prevalent neurodevelopmental disorder affecting children worldwide, with a significant impact on both the affected individuals and their families. The global prevalence of ADHD ranges from 5% to 7%, making it one of the most commonly diagnosed psychiatric disorders in childhood (1). The disorder is characterized by persistent patterns of inattention, hyperactivity, and impulsivity, leading to impairments in academic performance, social interactions, and overall functioning (2). The diagnosis of ADHD accords to the criteria described in the fifth edition of the Diagnostic and Statistical Manual of Mental Disorders (DSM-5) (3); however, diagnosing ADHD remains a complex task, often relying on subjective assessments such as parents' and teachers' reports, as well as clinical impressions from physicians. The ambiguity of relying solely on these subjective measures has led to concerns about the accuracy and reliability of ADHD diagnosis. There is a growing recognition that objective, neurophysiological measures could potentially provide valuable insights and complement the existing diagnostic procedures.

One such neurophysiological measure that has gained attention in recent years is the theta/beta ratio (TBR) derived from electroencephalography (EEG) recordings. The TBR reflects the ratio of theta to beta power in the brain's electrical activity, and has been proposed as a potential biomarker for ADHD (4). Several neurophysiological mechanisms underlie TBR and attention capacity. During the resting state, theta and alpha EEG bands dominate, shifting to the beta band during mental tasks (5). The elevated TBR in patients with ADHD may be linked to their difficulty in focusing on mental tasks. Some studies suggested that TBR could reflect cortical-subcortical interactions associated with inhibitory functioning, indicating the involvement of voluntary top-down processes during attentional control carried out by the dorsolateral prefrontal cortex (6). Therefore, the increased TBR observed in patients with ADHD may indicate either a deficient cortical response induced by mental effort or impaired voluntary top-down attention control. Support for these hypotheses is further substantiated by behavioral data. Zhang et al. (7) reported a positive correlation between frontal TBR and inattentive symptoms of ADHD. Additionally, Heinrich et al. (8) found that patients with higher TBR exhibited prolonged reaction times, indicating that difficulties in cortical response during mental tasks might manifest as increased TBR in this patient group. While TBR holds promise for aiding in ADHD diagnosis by offering an objective measure to supplement subjective assessments, its effectiveness in children with ADHD remains controversial. From a recent systemic review, the sensitivity and specificity of TBR on ADHD diagnosis varied from studies, casting doubt on the reliability and consistency of TBR as a biomarker for ADHD (9). Although the clinical application of TBR is highly controversial, it was approved by the US Food and Drug Administration as a confirmatory support for the diagnosis of ADHD in patients aged 6–17 years (10, 11).

Behavioral tests, such as the Conners Continuous Performance Test–3rd edition (CPT-3), have been employed to assess attentional capacity in individuals with ADHD (12). These tests provide additional objective measures, such as omissions, commissions, and hit reaction time, that can aid in the diagnostic process of ADHD (13). However, there are instances where CPT-3 results may contradict the clinical impression (14), further highlighting the complexity of ADHD diagnosis and the need for additional objective measures like TBR. Considering the ambiguity surrounding ADHD diagnosis and the potential of TBR and CPT-3 to provide objective measures, this study aims to investigate the correlation between TBR and CPT-3 scores in children with ADHD. We seek to examine the relationship between TBR and ADHD severity to better understand the clinical implications of these objective measures. By enhancing the diagnostic process with objective measures, we aim to improve the accuracy and reliability of ADHD diagnoses with neurophysiological biomarkers, such as TBR, and ultimately facilitate more effective interventions and treatment strategies for children with ADHD.

## 2 Material and methods

### 2.1 Participants

We conducted a retrospective study involving patients diagnosed with ADHD who were regularly seen for more than three times at the pediatric neurology or pediatric psychiatry clinic at Taipei Tzu Chi Hospital. The diagnosis of ADHD was established in accordance with DSM-5 criteria and assessed by experienced neurologist or psychiatrist. Additionally, we utilized the Swanson, Nolan, and Pelham (SNAP)-IV rating scales completed by parents and teachers to support the diagnosis. Because ADHD diagnosis typically does not necessitate EEG evaluation; consequently, patients with ADHD referred for EEG assessment often presented with other neurological manifestations such as seizures, tics, or headaches.

Between July 1, 2021, and December 31, 2022, we included 55 patients diagnosed with ADHD from 8 to 18 years old who underwent both EEG and CPT-3 assessments in our analysis. For CPT-3 T-scores, values exceeding 60 were considered abnormal across the nine variables, which include detectability, omission errors, commission errors, perseverations, hit reaction time (HRT), HRT standard deviation (SD), variability, HRT block change, and HRT inter-stimulus interval (ISI) change. Of the 55 ADHD patients, 41 exhibited one or more abnormal CPT-3 scores and were categorized into the ADHD with abnormal CPT-3 group, while the remaining 14 were placed into the ADHD with normal CPT-3 group. Additionally, we established a control group comprising 45 age-matched neurotypical individuals who received EEG studies due to headaches/migraines or syncope, but without ADHD symptoms. During CPT-3 examinations, all subjects were instructed to discontinue the use of ADHD medications, including methylphenidate. Thirty-three out of 55 ADHD patients were undergoing methylphenidate treatment at the time of EEG recording. Our study adhered to the ethical guidelines outlined in the Declaration of Helsinki and was granted approval by the Local Ethics Committee of Taipei Tzu Chi General Hospital (07-XD-095). Due to the retrospective nature of our study, written informed consent was waived.

### 2.2 EEG data processing

EEG recordings were acquired with Neurofax EEG-1200 (Nihon Kohden, Tokyo, Japan) using Ag/AgCl electrodes at a sampling rate of 200 Hz. A total of 19 electrodes were carefully placed on the scalp by a trained research technologist following the International 10–20 system, and all electrodes were referenced to the ground electrode located at the FPz position. After visual inspection of artifact rejection, a 10-s EEG recording during the eye-closed waking state was retrieved for further analysis. Digitized EEG data were processed offline using in-house MATLAB scripts developed for frequency analyses, which were transformed into frequency domains via a power spectral density (PSD) function (by Welch's method with the Hanning window, sampling rate at 200 Hz in a data block of 1 s, administered at a frequency resolution of 1 Hz and with half of the data overlap in each step), similar to our previous study (13, 15, 16). We calculated the normalized PSD at a specific frequency by measuring the ratio of the PSD of that specific frequency to the sum of the total PSD of 0–50 Hz, and analyzed in theta (4–7 Hz), alpha (8–12 Hz), and beta (13–25 Hz) frequency bands.

### 2.3 CPT-3

During the 14-min, 360-trial administration, participants were instructed to respond when any letter appeared on the screen, except for the non-target letter “X.” Specifically, they were asked to press the space bar or mouse button when a target letter was presented and to refrain from responding when the non-target letter “X” appeared. The CPT-3 is designed to assess attention-related issues in individuals aged 8 years and older. The CPT-3 provides scores for nine distinct variables, each of which serves as an indicator of various aspects of attention and response behavior. These variables include measures related to discriminative ability, errors of omission and commission, response speed, consistency in response speed, variability in response patterns across sub-blocks, and changes in response speed with different inter-stimulus intervals. These scores collectively offer a comprehensive evaluation of attention-related performance during the CPT-3, facilitating the identification and characterization of attention-related problems in individuals aged 8 years and older (17).

### 2.4 SNAP-IV scale

The Swanson, Nolan, and Pelham IV Scale (SNAP-IV) is a 26-item rating scale designed for evaluating ADHD and oppositional defiant disorder (ODD) symptoms in children between the ages of 6 and 18 years. The scale encompasses three subsets: attention deficit (9 items), hyperactivity/impulsivity disorder (9 items), and oppositional and defiance problems (8 items). Parents and teachers are enlisted to provide assessments, offering a more comprehensive understanding of the child's condition. Responses are recorded on a 4-point rating scale, ranging from 0 to 3 points, to indicate the extent to which the behavior is notably more frequent and severe when compared to typically developing children of the same age. The psychometric properties of the Chinese version of the SNAP-IV have been established, and it is widely employed in ADHD research within Taiwan (18, 19).

### 2.5 Statistical analysis

Statistical analyses were performed using SPSS version 19.0 (IBM Corporation, Armonk, NY, USA) for Windows (Microsoft Corporation, Redmond, WA, USA). Descriptive data are presented as mean ± SD. The chi-square test, Fisher's exact test, independent *t-*test, and ANOVA with Tukey analysis were used to compare factors such as age, sex, EEG indications, and TBR between patients with ADHD with abnormal CPT-3 scores, normal CPT-3 scores and control. Multivariate stepwise linear regression models were used to explore the relationship between variables, including TBR of Fz, Cz, and frontal electrodes, age, sex, diagnosis with or without ADHD, epilepsy, headache, tics, and syncope. Pearson's correlation analysis was used to assess the relationship between the TBR and the T-scores of CPT-3 variables. Two-sided *P* < 0.05 were considered to be statistically significant.

## 3 Results

### 3.1 Demographic and behavioral data of participants

The demographic information, CPT-3 T-scores, SNAP scale assessments from parents and teachers, as well as diagnosis other than ADHD, are presented in Table 1. The mean ages for the groups with ADHD and abnormal CPT-3 scores, ADHD and normal CPT-3 scores, and the control subjects were 10.3, 9.7, and 11.0 years old, respectively. Notably, there was a statistically significant difference in the distribution of sexes among these three groups. This discrepancy can be attributed to the fact that our control group, lacking ADHD symptoms, was drawn from patients interviewed in the neurology clinic. These individuals were frequently referred for EEG studies due to conditions like migraines, headaches, or syncope, which tend to be more prevalent in female adolescents.

**Table 1:** Demographic data, CPT-3 T scores and SNAP rating scales of patients with ADHD with normal CPT-3 scores, abnormal CPT-3 scores, and patients without ADHD.

	**ADHD/abnormal CPT-3 (*n =* 41)**	**ADHD/normal CPT-3 (*n =* 14)**	**Control (*n =* 45)**	***P*** **-value**		
				**Abnormal CPT-3 vs. normal CPT-3**	**Abnormal CPT-3 vs. control**	**Normal CPT-3 vs. control**
Age (SD)	10.3 (2.3)	9.7 (2.0)	11.0 (2.2)	0.823^a^	0.266 ^a^	0.208 ^a^
Sex (M/F)	30/11	13/1	19/26	0.156^b^	0.004^c^	0.001^c^
**CPT T score**						
Detectability (SD)	50.4 (11.7)	46.5 (7.8)	ND	0.169^d^		
Omission (SD)	53.0 (12.1)	45.3 (4.0)	ND	0.001^d^		
Commission (SD)	48.8 (10.2)	48.2 (6.7)	ND	0.799^d^		
Perseveration (SD)	54.7 (12.4)	48.2 (4.0)	ND	0.005^d^		
HRT (SD)	57.7 (11.3)	53.1 (4.6)	ND	0.038^d^		
HRT standard deviation (SD)	54.5 (10.2)	48.9 (3.9)	ND	0.004^d^		
Variability (SD)	52.3 (9.4)	49.5 (5.3)	ND	0.184^d^		
HRT block change (SD)	56.2 (11.9)	48.3 (6.3)	ND	0.003^d^		
HRT ISI change (SD)	57.5 (10.7)	51.1 (4.4)	ND	0.003^d^		
**SNAP scale of parents**	(*n =* 34)	(*n =* 13)				
ADD (SD)	16.4 (5.5)	17.5 (4.4)	ND	0.513^d^		
H/I (SD)	10.1 (7.3)	12.9 (6.6)	ND	0.245^d^		
**SNAP scale of teachers**	(*n =* 28)	(*n =* 10)				
ADD (SD)	11.4 (5.7)	16.6 (5.1)	ND	0.016^d^		
H/I (SD)	7.0 (7.3)	12.2 (6.2)	ND	0.051^d^		
**Diagnosis other than ADHD (%)**						
Seizure/epilepsy	10 (24.4)	2 (14.3)	1 (2.2)	0.709^c^	0.003^c^	0.137^c^
Headache/migraine	3 (7.3)	2 (14.3)	34 (75.6)	0.592^c^	< 0.001^c^	< 0.001^c^
Tics/Tourette	7 (17.1)	4 (28.6)	0 (0.0)	0.443^c^	0.004^c^	0.002^c^
Syncope	0 (0.0)	0 (0.0)	6 (13.3)	-	0.027^c^	0.319^c^

ADHD, attention deficit hyperactivity disorder; CPT-3, continuous performance test-3rd edition; HRT, hit reaction time; ISI, inter-stimulus interval; SNAP, Swanson, Nolan and Pelham; ADD, attention deficit disorder; H/I, hyperactivity/impulsivity. ^a^Analysis of variance with *post-hoc* Tukey analysis. ^b^Chi-squared test. ^c^Fisher's-exact test. ^d^Independent t-test.

In terms of CPT-3 variables, the group with ADHD and abnormal CPT-3 scores exhibited higher rates of omission, perseveration, HRT, HRT SD, HRT block change, and HRT ISI change. However, both the Attention Deficit Disorder (ADD) and Hyperactivity/Impulsivity (H/I) SNAP scale ratings from school teachers were higher in the group with ADHD and normal CPT-3 scores compared to the group with ADHD and abnormal CPT-3 scores.

### 3.2 TBR of eye-closed waking state

**Figure 1:**
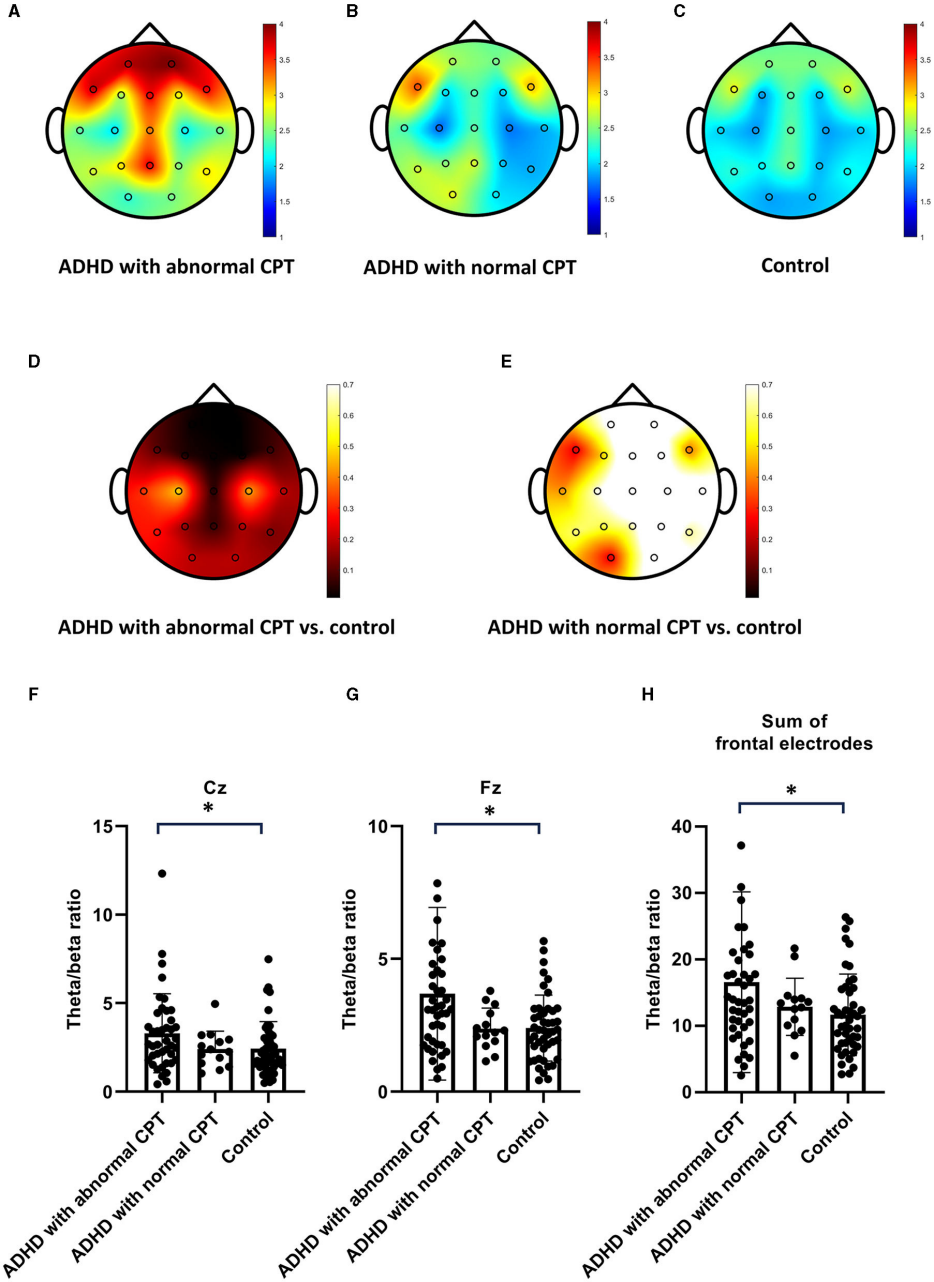
The theta/beta ratio (TBR) In patients with ADHD compared to the control group. **(A–C)** Present the TBR in ADHD patients with abnormal Continuous Performance Test−3rd edition (CPT-3), those with normal CPT-3, and the control group. ADHD patients with abnormal CPT exhibited higher TBR values on frontal electrodes. **(D, E)** Display the p-values indicating the significance of TBR differences between ADHD patients with abnormal CPT-3 and the control group, as well as between ADHD patients with normal CPT-3 and the control group. **(F–H)** Provide individual TBR values measured on Cz, Fz, and frontal electrodes for ADHD patients with abnormal CPT-3, those with normal CPT-3, and the control group.

Figures 1A–C depicted the TBR values recorded across all 19 electrodes for the three distinct groups. Noteworthy is that individuals in the ADHD with abnormal CPT-3 group exhibited markedly higher TBR levels on the frontal electrodes, Fz, Cz, and Pz in comparison to those in the control group (Figures 1A, C, D). Conversely, ADHD patients with normal CPT-3 scores displayed TBR levels similar to those observed in the control subjects (Figures 1B, E). No significant differences were observed in the TBR among ADHD patients undergoing methylphenidate treatment compared to those not receiving the treatment. In the next step, our objective was to identify a reliable biomarker for diagnosing ADHD. To accomplish this, we selected three distinct markers for consideration: Fz, Cz, and the aggregate total frontal TBR, which encompasses the summary values of F3, F4, Fz, F7, and F8. We presented individual TBR data for Cz, Fz, and a summary of the frontal electrodes (F3+F4+Fz+F7+F8) in Figures 1F–H. Despite a notable overlap in the data across these three groups, the ADHD with abnormal CPT-3 group consistently demonstrated higher TBR values at Cz, Fz, and the frontal electrodes.

Considering the potential influence of participant characteristics such as age, sex, the presence of ADHD, and other coexisting medical conditions on TBR, we conducted an exploration of the relationships between TBR values at Fz, Cz, and the frontal electrodes in conjunction with these variables using multivariate stepwise linear regression models. Following adjustments for these variables, the participant's age emerged as the most significant factor impacting TBR (Table 2). The presence of ADHD had a noteworthy effect on Fz TBR (*P* = 0.035), indicating a statistically significant association. However, its impact on Cz and frontal electrodes TBR showed only marginal significance (Table 2).

**Table 2:** Multivariate stepwise linear regression analysis coefficients for the association of Fz, Cz, and frontal electrodes TBR.

**Variables**	**Fz coefficient (*B*)**	**Fz *P*-value**	**Cz coefficient (*B*)**	**Cz *P*-value**	**Frontal electrodes coefficient (*B)***	**Frontal *P*-value**
Age	−0.38	< 0.001^**^	−0.43	< 0.001^**^	−0.431	< 0.001^**^
ADHD^a^	−0.20	0.035^*^	−0.15	0.098	−0.16	0.075
Sex^b^	−0.05	0.597	0.05	0.623	−0.08	0.414
Epilepsy^c^	0.11	0.251	−0.02	0.870	0.10	0.282
Headache^d^	−0.14	0.148	−0.12	0.209	−0.13	0.173
Tics^e^	−0.11	0.234	−0.16	0.090	−0.09	0.351
Syncope^f^	−0.33	0.730	−0.04	0.677	−0.03	0.723

^a^ADHD with abnormal CPT-3 = 0, ADHD with normal CPT-3 = 1, Control = 2. ^b^Male = 0, Female = 1. ^c^With epilepsy = 0, no epilepsy =1. ^d^With headache = 0, no headache = 1. ^e^With tics = 0, no tics = 1. ^f^With syncope = 0, no syncope = 1. TBR, theta/beta ratio; ADHD, attention deficit/hyperactivity disorder; CPT-3, continuous performance test – 3rd edition. ^*^P < 0.05, ^**^P < 0.001.

Given the observed differences in TBR values at Fz, Cz, and frontal electrodes across the three study groups, our subsequent investigation sought to discern any potential relationships between TBR and CPT-3 variables. However, our analyses did not reveal any significant correlations between TBR values at Fz, Cz, and frontal electrodes and the various CPT-3 variables, including detectability, omission errors, commission errors, perseverations, HRT, HRT SD, variability, HRT block change, and HRT ISI change (Table 3). Furthermore, we conducted an assessment of the correlations between TBR values and the SNAP-IV rating scales provided by both parents and teachers. However, similar to our previous findings, these analyses did not yield any significant correlations (Table 3).

**Table 3:** Pearson's correlations of TBR, CPT-3 variables, and SNAP-IV scales.

**CPT variables**	**TBR Fz**	**TBR Cz**	**TBR of frontal electrodes**
Detectability	−0.125	−0.076	−0.118
Omission	−0.013	0.047	−0.051
Commission	−0.133	−0.103	−0.111
Perseveration	−0.050	0.061	−0.099
HRT	0.181	0.121	0.138
HRT SD	−0.144	−0.099	−0.169
Variability	−0.161	−0.115	−0.155
HRT block change	0.011	−0.025	−0.050
HRT ISI change	−0.066	−0.018	−0.101
SNAP ADD parents	0.135	0.146	0.097
SNAP H/I parents	−0.006	0.015	−0.017
SNAP ADD teachers	−0.018	−0.058	0.112
SNAP H/I teachers	−0.254	−0.295	−0.194

TBR, theta/beta ratio; HRT, hit reaction time; ISI, inter-stimulus intervals; SNAP, Swanson, Nolan and Pelham; ADD, attention deficit disorder; H/I, hyperactivity/impulsivity.

Additionally, we attempted to establish a cutoff value for TBR that could potentially serve as a biomarker for ADHD. Regrettably, utilizing mean TBR + 1.5SD or TBR +1SD of Fz, Cz, and frontal electrodes as the threshold yielded sensitivities ranging from 7.3% to 14.5%. These low sensitivities pose a significant challenge to the clinical utility of these proposed biomarkers (Table 4).

**Table 4:** Sensitivity, specificity, PPV, and NPV of each TBR indicators.

**TBR indicators**	**Sensitivity**	**Specificity**	**PPV**	**NPV**
Fz + 1.5SD	7.3%	100%	100%	46.9%
Fz + 1SD	12.7%	95.6%	77.8%	47.3%
Cz + 1.5SD	7.3%	91.1%	50%	44.6%
Cz + 1SD	14.5%	91.1%	66.7%	46.6%
Frontal electrodes +1.5SD	7.3%	100%	100%	46.9%
Frontal electrodes +1SD	10.9%	93.3%	66.7%	46.2%

PPV, positive predictive value; NPV, negative predictive value; TBR, theta/beta ratio.

## 4 Discussion

In this study, we observed significant differences in TBR values among ADHD patients with abnormal and normal CPT-3 scores. Frontal/Cz/Fz TBR was elevated in ADHD children with abnormal CPT-3 scores, while TBR in ADHD children with normal CPT-3 was similar to controls. Stepwise linear regression revealed that age impacted TBR across Fz, Cz, and Frontal electrodes, while the diagnosis (ADHD) specifically influenced Fz. Despite TBR was potentially as an indicator of attentional capacity, as evaluated by CPT-3, there are no significant correlations between TBR values at any electrodes and CPT-3 variables or SNAP-IV rating scales by Pearson's correlation analysis. Additionally, no clear predictive utility was identified for a TBR cutoff. TBR values exhibited considerable overlap across groups, resulting in low sensitivity and negative predictive value when considering TBR as a potential neurophysiological biomarker for ADHD. These findings underscore the complexity of utilizing TBR as a standalone biomarker and emphasize the necessity of considering multiple factors in the assessment of attention-related conditions.

ADHD is a neurodevelopmental disorder characterized by persistent patterns of inattention, hyperactivity, and impulsivity. The underlying neuromechanism of ADHD is complex and involves various regions of the brain (20). Traditionally, the frontal lobe was considered to play a pivotal role, given its responsibility for executive functions, which encompass a range of cognitive processes like attention control, inhibition, working memory, and cognitive flexibility (21–24). However, other brain areas, including the basal ganglia, corpus callosum, temporal lobes, and caudate nuclei, have also been reported to show abnormalities in patients with ADHD (20). In our study, the most prominent differences in TBR between patients with ADHD and control subjects were observed in the frontal head regions, which underscores the notion that ADHD involves some form of frontal lobe dysfunction. Many previous studies predominantly focused on calculating the TBR at Cz as a diagnostic tool to differentiate between individuals with ADHD and control subjects. However, the reported results varied, potentially due to Cz TBR not consistently representing the largest difference between these two groups. Snyder et al. conducted a study with 97 ADHD patients and 62 non-ADHD control subjects aged between 6 and 18 years. They observed a significant increase in Cz TBR in ADHD patients compared to control subjects, achieving a diagnostic sensitivity of 87% and specificity of 94% (25). However, Loo et al. reported no significant differences in Cz TBR between ADHD and control groups in children or adolescents and even found a decreased TBR in adult ADHD patients (26). This finding contradicts another study by Kiiski et al. (27), which reported that Cz TBR failed to identify ADHD status in adult patients. Studies utilizing Fz TBR as an EEG biomarker for ADHD are scarce. In a recent analysis of QEEG data from two large-scale studies, the ICAN study and the iSPOT-A study, it was concluded that TBR cannot reliably distinguish ADHD from controls. However, differences were more pronounced at Fz than Cz, resulting in marginal significance (*P*-value between 0.062 to 0.101) (28). Furthermore, in our study, we observed distinct TBR patterns in ADHD patients with and without abnormal CPT-3 scores. This suggests that TBR may reflect individual attention capacity similarly to behavioral exams. Given the heterogeneous etiologies and presentations of ADHD, utilizing TBR as a biomarker for all ADHD patients could potentially dilute its effectiveness as an indicator for distinguishing them from control subjects. This outcome brings out the complexity of ADHD's neurophysiological mechanisms and highlights the need for larger-scale studies in the future.

An intriguing finding emerged from this study, particularly in the cohort of ADHD patients receiving consistent clinical follow-up from experienced physicians. It was noted that individuals with abnormal CPT-3 scores demonstrated elevated TBR levels. Contrarily, when evaluated by school teachers using the SNAP-IV scales, patients with normal CPT-3 scores exhibited higher ratings. This dual observation adds a layer of complexity to our understanding of ADHD, suggesting that distinct assessment methods may yield varying insights into the condition. Within our cohort, 14 out of 55 patients diagnosed with ADHD (25.5%) exhibited normal CPT-3 scores, indicating a low sensitivity of CPT-3 in the diagnosis of ADHD. This finding aligns with prior research which has highlighted that behavioral assessments for ADHD, including tools like CPT-3, tend to demonstrate high specificity but low sensitivity in ADHD diagnosis (12, 29). Regarding the potential use of TBR as a diagnostic tool for ADHD, while a review article by Clarke et al. reported sensitivity levels ranging from 46.9% to 94% (9), there is also a report indicating the inability to differentiate between ADHD and control subjects using Cz TBR (30). In our study, while TBR exhibited distinctions between patients with ADHD and control subjects, it demonstrated low sensitivity and high specificity as a potential biomarker for diagnosing ADHD. Because TBR can serve as a reflection of neurophysiological processes underlying attention and potentially represent an individual's attentional capacity—measurable through behavioral assessments like CPT-3—it's not surprising that elevated TBR was only evident in ADHD patients with abnormal CPT-3 scores. However, it's crucial to note that the most profound impact of ADHD lies in the disruptive symptoms that significantly impaired daily functioning. These include difficulties in organizing tasks and activities, as well as tendencies to interrupt or intrude on others. These behavioral challenges can substantially hinder peer relationships, academic or work performance, and may lead to long-term consequences for individuals affected by ADHD (2, 31). Our findings reinforce the notion that utilizing measures of attention capacity, whether through TBR or CPT-3, is not alone sufficient for an accurate ADHD diagnosis. Clinical expertise and impressions from experienced physicians, as well as the assessment of daily symptoms by caregivers and teachers, hold equal in this diagnostic process.

Our study had several limitations. Firstly, due to the retrospective design, there were variations in sex and underlying neurological conditions between patients with ADHD and control subjects. Additionally, the absence of a 'clean' ADHD and control subjects without any other neurological conditions may pose challenges to the generalization of our results. However, after conducting a multivariate stepwise linear regression analysis to account for these factors, we still observed increased TBR in ADHD patients with abnormal CPT-3 scores. Secondly, we did not have CPT-3 data for control subjects; consequently, the correlations between TBR and behavioral data were only examined in ADHD patients. To draw definitive conclusions regarding the utility of EEG and TBR in ADHD diagnosis, a prospective study with a larger cohort, including both patients and control subjects, and comprehensive neuropsychometric evaluations alongside EEG, is warranted. Such an approach would provide a more robust foundation for assessing the diagnostic potential of EEG and TBR in ADHD.

## 5 Conclusion

In conclusion, our study observed the utility of both TBR and CPT-3 in evaluating attentional capacity in individuals; however, these assessments demonstrated low sensitivity in diagnosing ADHD. The comprehensive evaluation, incorporating clinical expertise, input from parents, and detailed neuropsychometric tests, remains crucial for achieving a thorough and accurate understanding of ADHD. This highlights the necessity for a holistic approach in clinical practice when addressing this complex neurodevelopmental condition.

## Data availability statement

The raw data supporting the conclusions of this article will be made available by the authors, without undue reservation.

## Ethics statement

The studies involving humans were approved by the Institutional Review Board of Taipei Tzu Chi Hospital. The studies were conducted in accordance with the local legislation and institutional requirements. Written informed consent for participation was not required from the participants or the participants' legal guardians/next of kin in accordance with the national legislation and institutional requirements.

## Author contributions

T-SW: Conceptualization, Data curation, Writing—original draft, Writing—review & editing. S-SW: Data curation, Formal analysis, Software, Writing—review & editing. C-LW: Data curation, Formal analysis, Software, Writing—review & editing. S-BW: Conceptualization, Funding acquisition, Writing—original draft, Writing—review & editing.

## References

[1] Sayal K Prasad V Daley D Ford T Coghill D. ADHD in children and young people: prevalence, care pathways, and service provision. Lancet Psychiatry. (2018) 5:175–86. 10.1016/S2215-0366(17)30167-0 29033005

[2] Faraone SV Asherson P Banaschewski T Biederman J Buitelaar JK Ramos-Quiroga JA. Attention-deficit/hyperactivity disorder. Nat Rev Dis Primers. (2015) 1:15020. 10.1038/nrdp.2015.20 27189265

[3] APA. Diagnostic and Statistical Manual of Mental Disorders, 5th Edn. Washington, DC: APA (2013).

[4] Barry RJ Clarke AR Johnstone SJ. A review of electrophysiology in attention-deficit/hyperactivity disorder: I. Q. Quant Electroencephal Clin Neurophysiol. (2003) 114:171–83. 10.1016/S1388-2457(02)00362-0 12559224

[5] Clarke AR Barry RJ Karamacoska D Johnstone SJ. The EEG theta/beta ratio: a marker of arousal or cognitive processing capacity? Appl Psychophysiol Biofeedback. (2019) 44:123–9. 10.1007/s10484-018-09428-6 30604100

[6] van Son D De Blasio FM Fogarty JS Angelidis A Barry RJ Putman P. Frontal EEG theta/beta ratio during mind wandering episodes. Biol Psychol. (2019) 140:19–27. 10.1016/j.biopsycho.2018.11.003 30458199

[7] Zhang D-W Li H Wu Z Zhao Q Song Y Liu L. Electroencephalogram theta/beta ratio and spectral power correlates of executive functions in children and adolescents with AD/HD. J Atten Disord. (2019) 23:721–32. 10.1177/1087054717718263 28689463

[8] Heinrich H Busch K Studer P Erbe K Moll GH Kratz O. Spectral analysis of attention in ADHD: implications for neurofeedback training? Front Hum Neurosci. (2014) 8:611. 10.3389/fnhum.2014.00611 25191248 PMC4139738

[9] Clarke AR Barry RJ Johnstone S. Resting state EEG power research in attention-deficit/hyperactivity disorder: a review update. Clin Neurophysiol. (2020) 131:1463–79. 10.1016/j.clinph.2020.03.029 32387965

[10] Saad JF Kohn MR Clarke S Lagopoulos J Hermens DF. Is the theta/beta EEG marker for ADHD inherently flawed? J Atten Disord. (2018) 22:815–26. 10.1177/1087054715578270 25823742

[11] Gloss D Varma JK Pringsheim T Nuwer MR. Practice advisory: The utility of EEG theta/beta power ratio in ADHD diagnosis. Rep Guideline Dev Dissem Impl Subcomm Am Acad Neurol. (2016) 87:2375–9. 10.1212/WNL.0000000000003265 27760867 PMC5135022

[12] Ord AS Miskey HM Lad S Richter B Nagy K Shura RD. Examining embedded validity indicators in Conners continuous performance test-3 (CPT-3). The Clin Neuropsychol. (2021) 2:1–16. 10.1080/13854046.2020.1751301 32364040

[13] Tsai LP Wang SS Chee SY Wong SB. Dynamic changes in the quantitative electroencephalographic spectrum during attention tasks in patients with prader-willi syndrome. Front Genet. (2022) 13:763244. 10.3389/fgene.2022.763244 35368678 PMC8965856

[14] McGee RA Clark SE Symons DK. Does the conners' continuous performance test aid in ADHD diagnosis? J Abnorm Child Psychol. (2000) 28:415–24. 10.1023/A:1005127504982 11100916

[15] Pan MK Li YS Wong SB Ni CL Wang YM Liu WC. Cerebellar oscillations driven by synaptic pruning deficits of cerebellar climbing fibers contribute to tremor pathophysiology. Sci Transl Med. (2020) 12:1769. 10.1126/scitranslmed.aay1769 31941824 PMC7339589

[16] Wong SB Wang YM Lin CC Geng SK Vanegas-Arroyave N Pullman SL. Cerebellar oscillations in familial and sporadic essential tremor. Cerebellum. (2022) 21:425–31. 10.1007/s12311-021-01309-9 34341893 PMC8970339

[17] Keith Conners C Sitarenios G Ayearst L. Conners' continuous performance test. Encycl Clin Neuropsychol. (2018) 12:1535. 10.1007/978-3-319-57111-9_1535

[18] Gau SS Shang CY Liu SK Lin CH Swanson JM Liu YC. Psychometric properties of the Chinese version of the Swanson, Nolan, and Pelham, version IV scale - parent form. Int J Methods Psychiatr Res. (2008) 17:35–44. 10.1002/mpr.237 18286459 PMC6878250

[19] Gau SS-F Lin C-H Hu F-C Shang C-Y Swanson JM Liu Y-C. Psychometric properties of the chinese version of the Swanson, Nolan, and Pelham, version IV scale-teacher form. J Pediatr Psychol. (2008) 34:850–61. 10.1093/jpepsy/jsn133 19074488

[20] Firouzabadi FD Ramezanpour S Firouzabadi MD Yousem IJ Puts NAJ Yousem DM. Neuroimaging in attention-deficit/hyperactivity disorder: recent advances. AJR Am J Roentgenol. (2022) 218:321–32. 10.2214/AJR.21.26316 34406053

[21] Morecraft RJ Geula C Mesulam MM. Architecture of connectivity within a cingulo-fronto-parietal neurocognitive network for directed attention. Arch Neurol. (1993) 50:279–84. 10.1001/archneur.1993.00540030045013 8442707

[22] Filipek PA Semrud-Clikeman M Steingard RJ Renshaw PF Kennedy DN Biederman J. Volumetric MRI analysis comparing subjects having attention-deficit hyperactivity disorder with normal controls. Neurology. (1997) 48:589–601. 10.1212/WNL.48.3.589 9065532

[23] Petersen SE Posner MI. The attention system of the human brain: 20 years after. Annu Rev Neurosci. (2012) 35:73–89. 10.1146/annurev-neuro-062111-150525 22524787 PMC3413263

[24] Friedman NP Robbins TW. The role of prefrontal cortex in cognitive control and executive function. Neuropsychopharmacology. (2022) 47:72–89. 10.1038/s41386-021-01132-0 34408280 PMC8617292

[25] Snyder SM Quintana H Sexson SB Knott P Haque AF Reynolds DA. Blinded, multi-center validation of EEG and rating scales in identifying ADHD within a clinical sample. Psychiatry Res. (2008) 159:346–58. 10.1016/j.psychres.2007.05.006 18423617

[26] Loo SK Cho A Hale TS McGough J McCracken J Smalley SL. Characterization of the theta to beta ratio in ADHD: identifying potential sources of heterogeneity. J Atten Disord. (2013) 17:384–92. 10.1177/1087054712468050 23264365

[27] Kiiski H Bennett M Rueda-Delgado LM Farina FR Knight R Boyle R. EEG spectral power, but not theta/beta ratio, is a neuromarker for adult ADHD. Eur J Neurosci. (2020) 51:2095–109. 10.1111/ejn.14645 31834950

[28] van Dijk H deBeus R Kerson C Roley-Roberts ME Monastra VJ Arnold LE. Different spectral analysis methods for the theta/beta ratio calculate different ratios but do not distinguish ADHD from controls. Appl Psychophysiol Biofeedback. (2020) 45:165–73. 10.1007/s10484-020-09471-2 32436141 PMC7391403

[29] Scimeca LM Holbrook L Rhoads T Cerny BM Jennette KJ Resch ZJ. Examining Conners continuous performance test-3 (CPT-3) embedded performance validity indicators in an adult clinical sample referred for ADHD evaluation. Dev Neuropsychol. (2021) 46:347–59. 10.1080/87565641.2021.1951270 34256665

[30] Liechti MD Valko L Müller UC Döhnert M Drechsler R Steinhausen H-C. Diagnostic value of resting electroencephalogram in attention-deficit/hyperactivity disorder across the lifespan. Brain Topogr. (2013) 26:135–51. 10.1007/s10548-012-0258-6 23053601

[31] Harpin V Mazzone L Raynaud JP Kahle J Hodgkins P. Long-term outcomes of ADHD: a systematic review of self-esteem and social function. J Atten Disord. (2016) 20:295–305. 10.1177/1087054713486516 23698916

